# Near-Complete Genome Sequences of Nine SARS-CoV-2 Strains Harboring the D614G Mutation in Malaysia

**DOI:** 10.1128/MRA.00657-21

**Published:** 2021-08-05

**Authors:** Ummu Afeera Zainulabid, Norhidayah Kamarudin, Ahmad Hafiz Zulkifly, Han Ming Gan, Darren Dean Tay, Shing Wei Siew, Aini Syahida Mat Yassim, Sharmeen Nellisa Soffian, Ahmad Afif Mohd Faudzi, Ahmad Mahfuz Gazali, Gaanty Pragas Maniam, Hajar Fauzan Ahmad

**Affiliations:** a Department of Internal Medicine, Sultan Ahmad Shah Medical Centre @IIUM, Kuantan, Pahang, Malaysia; b Microbiology Unit, Department of Pathology & Laboratory Medicine, Sultan Ahmad Shah Medical Centre @IIUM, Kuantan, Pahang, Malaysia; c GeneSeq Sdn. Bhd., Bandar Baru Bukit Beruntung, Selangor, Malaysia; d Australian Institute for Bioengineering and Nanotechnology, The University of Queensland, St. Lucia, QLD, Australia; e College of Engineering, Universiti Malaysia Pahang, Lebuhraya Tun Razak, Gambang, Pahang, Malaysia; f Centre of Excellence for Data Science and Artificial Intelligence (Data Science Centre), Universiti Malaysia Pahang, Lebuhraya Tun Razak, Gambang, Pahang, Malaysia; g Faculty of Industrial Sciences & Technology, Universiti Malaysia Pahang, Lebuhraya Tun Razak, Gambang, Pahang, Malaysia; h Centre for Research in Advanced Tropical Bioscience (Biotropic Centre), Universiti Malaysia Pahang, Lebuhraya Tun Razak, Gambang, Pahang, Malaysia; DOE Joint Genome Institute

## Abstract

Here, we report the nearly complete genome sequences of nine severe acute respiratory syndrome coronavirus 2 (SARS-CoV-2) variants with the D614G mutation. These viruses were detected from various infected individuals with different levels of severity from Pahang, Malaysia. In addition, this study described the presence of lineage B.1.351 as a type of variant of concern (VOC) and lineages B.1.466.2 and B.1.524 as local variants.

## ANNOUNCEMENT

The current pandemic of coronavirus disease 19 (COVID-19) is caused by severe acute respiratory syndrome coronavirus 2 (SARS-CoV-2), which belongs to the viral family *Coronaviridae* and genus *Betacoronavirus* (1). The COVID-19 D614G mutation was associated with higher risk of infection (2). Here, we report nine nearly complete genome sequences of variants of concern (VOC) from the Beta B.1.351 lineage and several unassigned variants that belong to local lineages (3). The clinical specimens of nine patients with various clinical presentations in Sultan Ahmad Shah Medical Centre @IIUM (SASMEC @IIUM) were collected directly from combined oropharyngeal and nasopharyngeal swabs in April 2020 and April 2021. These individuals were detected as COVID-19-infected individuals through reverse transcriptase PCR (RT-PCR) (threshold cycle [*C_T_*] value, <30) and traced from active contact tracing during severe acute respiratory infection (SARI) surveillance. The study was approved by the International Islamic University Malaysia Research Ethics Committee (IREC 2021-080).

The total RNA was extracted using a Maxwell HT simplyRNA kit (Promega, USA) and converted into cDNA using SuperScript IV reverse transcriptase (Invitrogen) with some modifications; a hexamer annealing and extension step of 25°C for 2 min was performed, followed by cDNA synthesis at 42°C for 50 min. A portion (1:10 volume) of the cDNA from sample IIUM91 was used as the template for multiplex PCR using Q5 high-fidelity DNA polymerase (New England BioLabs [NEB], USA) and the Artic v3 primer pools. The amplicons for more recent samples were generated using the commercially available NEBNext ARTIC SARS-CoV-2 companion kit (NEB). Equal volumes of PCR products obtained from the two primer pools were mixed; pool 1 and pool 2 were mixed according to the designated protocols (4) and purified using AMPure XP for PCR purification (Beckman Coulter Life Sciences, USA). The purified PCR products were quantified using a double-stranded DNA (dsDNA) high-sensitivity assay (DeNovix, Inc., USA), and 50 ng was used to construct an Illumina library using the NEB UltraII library preparation kit as described previously (5). The constructed library was sequenced on an iSeq 100 sequencing system (Illumina, Inc., USA) with run configuration of 1 × 300 bp or 1 × 250 bp. On average, 125,319 single-end reads were generated from each sample (minimum, 68,787; maximum, 423,112). These raw reads were used to reconstruct the SARS-CoV-2 genome using a combination of bioinformatic tools as listed at https://github.com/CDCgov/SARS-CoV-2_Sequencing/tree/master/protocols/BFX-UT_ARTIC_Illumina. Briefly, the raw reads were aligned to the reference strain WuHan-Hu-1 genome (GenBank accession number MN908947) using the Burrows-Wheeler Aligner MEM algorithm (BWA-MEM) v0.7.17-r1188 (6) and subsequently trimmed to remove the primer binding region, and a consensus genome was generated from the filtered alignment using iVar v1.2.2 (7). Details regarding the reported genomes are summarized in Table 1.

**TABLE 1:** Summary of SARS-CoV-2 strains sequenced from Pahang, Malaysia

Sequence name	Genome size (bp)	Lineage	WHO label	Clade^*b*^	GC content (%)	No. of reads	BioSample no.
hCoV-19/Malaysia/UMP-IIUM5480/2021	29,764	B.1.351	Beta (β)	GH	38.0	91,342	SAMN19778019
hCoV-19/Malaysia/IIUM5556/2021	29,764	B.1.351	Beta (β)	GH	37.9	113,985	SAMN19778013
hCoV-19/Malaysia/IIUM5676/2021	29,782	B.1.524	No WHO label; Malaysian lineage^*a*^	G	38.0	83,614	SAMN19778016
hCoV-19/Malaysia/IIUM5754/2021	29,764	B.1.351	Beta (β)	GH	38.0	104,861	SAMN19778014
hCoV-19/Malaysia/IIUM5755/2021	29,764	B.1.351	Beta (β)	GH	38.0	83,456	SAMN19778015
hCoV-19/Malaysia/IIUM5763/2021	29,782	B.1.466.2	No WHO label; Indonesian lineage^*a*^	GH	37.9	68,787	SAMN19778017
hCoV-19/Malaysia/IIUM5770/2021	29,764	B.1.351	Beta (β)	GH	38.0	70,583	SAMN19778018
hCoV-19/Malaysia/IIUM6472/2021	29,764	B.1.351	Beta (β)	GH	37.9	88,134	SAMN19778020
hCoV-19/Malaysia/IIUM91/2020	29,701	B.1.468	No WHO label; Indonesia/Singapore lineage^*a*^	GH	38.0	423,112	SAMN16383837

a These lineages were listed as neither variants of concern (VOC) nor variants of interest (VOI) by the World Health Organization. We determined these lineages using the PANGO Web server (3), which is available online at https://cov-lineages.org/index.html.

b Members of clade G contain mutations C241T, C3037T, and A23403G, as well as S-D614G; members of clade GH contain mutations C241T, C3037T, A23403G, and G25563T, as well as S-D614G plus NS3-Q57H.

Of the nine strains sequenced, six were classified as the B.1.351 variant, which harbors the E484K and N501Y mutations commonly associated with increased transmission rate (8). In addition, this study also documented B.1.524 of Malaysian lineage and B.1.466.2 and B.1.468 of Indonesian lineages.

### Data availability.

These sequences were deposited in GenBank under the accession numbers MW079428.1 and MZ443817.1 to MZ443824.1. The accession numbers in the NCBI Sequence Read Archive (SRA) are SRP286590 and SRP324679. The sequences in the GISAID database are as follows: EPI_ISL_455313, EPI_ISL_2622006, EPI_ISL_2622007, EPI_ISL_2622045, EPI_ISL_2622046, EPI_ISL_2622047, EPI_ISL_2622079, EPI_ISL_2622088, and EPI_ISL_2622089.

## References

[1] Gorbalenya AE, Baker SC, Baric RS, de Groot RJ, Drosten C, Gulyaeva AA, Haagmans BL, Lauber C, Leontovich AM, Neuman BW, Penzar D, Perlman S, Poon LLM, Samborskiy DV, Sidorov IA, Sola I, Ziebuhr J, Coronaviridae Study Group of the International Committee on Taxonomy of Viruses. 2020. The species severe acute respiratory syndrome-related coronavirus: classifying 2019-nCoV and naming it SARS-CoV-2. Nat Microbiol 5:536–544. doi:10.1038/s41564-020-0695-z. 32123347 PMC7095448

[2] Syahida Mat Yassim A, Fazli Farida Asras M, Mahfuz Gazali A, Marcial-Coba MS, Afeera Zainulabid U, Fauzan Bin Ahmad H. 2021. COVID-19 outbreak in Malaysia: decoding D614G mutation of SARS-CoV-2 virus isolated from an asymptomatic case in Pahang. Mater Today Proc doi:10.1016/j.matpr.2021.02.387. PMC7914017 33680867

[3] O’Toole Á, Scher E, Underwood A, Jackson B, Hill V, McCrone JT, Colquhoun R, Ruis C, Abu-Dahab K, Taylor B, Yeats C, Du Plessis L, Maloney D, Medd N, Attwood SW, Aanensen DM, Holmes EC, Pybus OG, Rambaut A. 2021. Assignment of epidemiological lineages in an emerging pandemic using the Pangolin tool. Virus Evol doi:10.1093/ve/veab064. PMC8344591 34527285

[4] Quick J. 2020. nCoV-2019 sequencing protocol. https://www.protocols.io/view/ncov-2019-sequencing-protocol-bbmuik6w.

[5] Glenn TC, Nilsen RA, Kieran TJ, Sanders JG, Bayona-Vásquez NJ, Finger JW, Pierson TW, Bentley KE, Hoffberg SL, Louha S, Garcia-De Leon FJ, del Rio Portilla MA, Reed KD, Anderson JL, Meece JK, Aggrey SE, Rekaya R, Alabady M, Belanger M, Winker K, Faircloth BC. 2019. Adapterama I: universal stubs and primers for 384 unique dual-indexed or 147,456 combinatorially-indexed Illumina libraries (iTru & iNext). PeerJ 7:e7755. doi:10.7717/peerj.7755. 31616586 PMC6791352

[6] Wu F, Zhao S, Yu B, Chen Y-M, Wang W, Song Z-G, Hu Y, Tao Z-W, Tian J-H, Pei Y-Y, Yuan M-L, Zhang Y-L, Dai F-H, Liu Y, Wang Q-M, Zheng J-J, Xu L, Holmes EC, Zhang Y-Z. 2020. A new coronavirus associated with human respiratory disease in China. Nature 579:265–269. doi:10.1038/s41586-020-2008-3. 32015508 PMC7094943

[7] Grubaugh ND, Gangavarapu K, Quick J, Matteson NL, De Jesus JG, Main BJ, Tan AL, Paul LM, Brackney DE, Grewal S, Gurfield N, Van Rompay KKA, Isern S, Michael SF, Coffey LL, Loman NJ, Andersen KG. 2019. An amplicon-based sequencing framework for accurately measuring intrahost virus diversity using PrimalSeq and iVar. Genome Biol 20:8. doi:10.1186/s13059-018-1618-7. 30621750 PMC6325816

[8] Tegally H, Wilkinson E, Giovanetti M, Iranzadeh A, Fonseca V, Giandhari J, Doolabh D, Pillay S, San EJ, Msomi N, Mlisana K, von Gottberg A, Walaza S, Allam M, Ismail A, Mohale T, Glass AJ, Engelbrecht S, Van Zyl G, Preiser W, Petruccione F, Sigal A, Hardie D, Marais G, Hsiao M, Korsman S, Davies M-A, Tyers L, Mudau I, York D, Maslo C, Goedhals D, Abrahams S, Laguda-Akingba O, Alisoltani-Dehkordi A, Godzik A, Wibmer CK, Sewell BT, Lourenço J, Alcantara LCJ, Pond SLK, Weaver S, Martin D, Lessells RJ, Bhiman JN, Williamson C, de Oliveira T. 2020. Emergence and rapid spread of a new severe acute respiratory syndrome-related coronavirus 2 (SARS-CoV-2) lineage with multiple spike mutations in South Africa. medRxiv 2020.12.21.20248640.

